# miR-1266-3p Suppresses Epithelial-Mesenchymal Transition in Colon Cancer by Targeting P4HA3

**DOI:** 10.1155/2022/1542117

**Published:** 2022-04-07

**Authors:** Hailang Zhou, Shu Huang, Changjiang Shao, Junwei Zou, Aijun Zhou, Jiufeng Yu, Chunfang Xu

**Affiliations:** ^1^Department of Gastroenterology, The First Affiliated Hospital of Soochow University, Suzhou, Jiangsu 215006, China; ^2^Department of Gastroenterology, Lianshui People's Hospital Affiliated to Kangda College of Nanjing Medical University, Huaian, Jiangsu 223400, China; ^3^Department of Gastroenterology, The Second People's Hospital of Lianyungang, Lianyungang, Jiangsu 222006, China; ^4^Department of General Surgery, The Second Affiliated Hospital of Wannan Medical College, Wuhu, Anhui 241000, China; ^5^Department of Traditional Chinese Medicine, Lianshui People's Hospital Affiliated to Kangda College of Nanjing Medical University, Huaian, Jiangsu 223400, China

## Abstract

Numerous studies have been conducted to demonstrate that miRNA is strongly related to colon cancer progression. Nevertheless, there are few studies regarding the function for miR-1266-3p in colon cancer, and the molecular mechanism remains poorly know. Our study was designed to examine the level of miR-1266-3p expression among the colon cancer tissue and cell and to study the role and regulatory mechanism for miR-1266-3p among colon cancer's malignant biologic behavior. First, we found that miR-1266-3p expression was distinctly lower in colonic carcinoma tissues and cells than in nontumor ones, and the prognosis of low miR-1266-3p patients was distinctly worse than that of high miR-1266-3p patients. Second, we predicted that the target gene of miR-1266-3p was prolyl 4-hydroxylase subunit alpha 3 (P4HA3) through bioinformatics, and the targeting relationship between the two was verified by a dual luciferase assay report. Furthermore, miR-1266-3p inhibited the growth and metastasis of colon cancer in vitro as well as in vivo, and this effect could be alleviated by overexpressing P4HA3. Even more importantly, our study demonstrated that miR-1266-3p inhibited epithelial-mesenchymal transition (EMT) by targeting P4HA3. In conclusion, miR-1266-3p could inhibit growth, metastasis, and EMT in colon cancer by targeting P4HA3. Our discoveries might offer a novel target for colon cancer diagnosis and treatment.

## 1. Introduction

Colon cancer ranks third among the most commonly diagnosed cancers worldwide, and is the second leading cause of death from cancer, particularly in developed countries [1]. Each year, over 1.9 million people get this cancer with up to more than 900 thousand dying from it [2]. Since 2000, rates of incidence and mortality due to colon cancer in young people under the age of 50 have increased very rapidly [3]. Colon cancer progression occurs on the basis of various mechanisms, involving oncogenic gene activation and suppression of gene inactivation [4]. Accordingly, in-depth understanding of colon carcinogenesis molecular mechanism is essential, as well as the study of novel diagnostic and therapeutic markers for colon cancer.

microRNAs (miRNAs) are a group of small (about 19-23 nucleotides), noncoding, single-stranded RNA molecules which exert significant regulation at the posttranscriptional level [5, 6]. A growing number of studies reveal that miRNAs act as an essential part in facilitating or suppressing tumor growth and metastasis through modulation of oncogenes or repressor genes [7–9]. There have been studies to identify that miRNAs are differentially expressed in colon carcinoma. They have been strongly associated with the biologic and clinical features in colon cancer and act as key roles in the pathogenesis of colon cancer [10, 11]. For instance, miR-378 restrains colon cancer growth and metastasis via suppressing SDAD1 [12]. Another study demonstrated that miR-204-3p inhibits colon cancer growth and metastasis by inhibiting HMGA2 [13]. Several other studies have revealed that miRNAs promote the malignant biological behaviors of colon cancer. For example, miR-937 has been dramatically upregulated in colon cancer and promotes colon cancer growth and metastasis [14]. Another study showed that miR-3120-5p facilitates colon cancer stem cell properties and invasion through downregulating Axin2 [15]. With these findings, miRNAs are now becoming a strategic focus point used to deal with colon cancer. As an important member of miRNAs, miR-1266-3p is known to perform an essential function in multiple types of cancer, such as prostate cancer [16], cervical squamous cancer [17], pancreatic cancer [18], papillary thyroid cancer [19], and gastric cancer [20]. Yet, miR-1266-3p's role in colon cancer is not reported before.

Prolyl 4-hydroxylase (P4H), one evolutionarily well-preserved enzyme, is made up by two *α* and two *β* subunits. P4H has been characterized to have three kinds of *α*-subunit. P4HA1 gene encodes *α* (I), P4HA2 gene encodes *α* (II), P4HA3 gene encodes *α* (III) [21]. Multiple researches have been conducted to report the correlation on P4HA3 with various cancers. The previous research found that the expression levels of both P4HA3 mRNA and protein of gastric cancer tissues were obviously increased compared with nongastric cancer tissues [22]. Also, P4HA3 manifested a cancer promoter through the regulation of EMT in the head and neck squamous cell cancer [23]. Additional study has indicated that P4HA3 and COL6A6 jointly restrain pituitary adenoma progression through obstructing PI3K-Akt signaling [24]. However, whether P4HA3 can be regulated by miRNAs in cancer, especially in colon cancer, is unclear.

In the current research, it was found that miR-1266-3p played an anticancer role in colon cancer. Our work also demonstrated that P4HA3 is a direct target gene of miR-1266-3p and that it could reverse the inhibitory effect of miR-1266-3p on colon cancer growth and metastasis. More importantly, we found that miR-1266-3p could inhibit EMT in colon cancer by targeting P4HA3. Together, all the results imply that miR-1266-3p may be a potential diagnostic and therapeutic target for colon cancer.

## 2. Methods and Materials

### 2.1. Bioinformatic Analysis Based on the Database

The data downloaded from TCGA database and GEO database was processed with Ualcan online analysis software (http://ualcan.path.uab.edu/index.html) and R software (R version 4.0.5). The overall survival prognostic value of miR-1266-3p in colon cancer was further assessed by Kaplan Meier plotter (https://kmplot.com/analysis/).

### 2.2. Clinical Samples

There were 5 pairs of colon cancer and paraneoplastic tissue from Lianshui People's Hospital (Huai'an, China). All colon cancer patients signed an informed written consent form and then acted in accordance with the Declaration of Helsinki. All patients had not received preoperative radiotherapy or chemotherapy. Patients with inflammatory diseases, polygenic cancers, or automatic immune diseases were not included.

### 2.3. Cell Culture

NCM460, a colon normal epithelium line, was obtained from BNCC (Beijing, China). Colonic carcinoma cell line SW1116 was taken from Zhongqiao Xinzhou Bio-Technology Co., Ltd. (Shanghai, China), and the other four colon cancer cell lines, HCT116, HT29, SW480, and SW620, were taken from the NCAC (Shanghai, China). SW1116 was cultured in Leibovitz's L-15 medium, NCM460 in DMEM medium, and the other four colon cancer cell lines in RPMI-1640 medium. DMEM medium and RPMI-1640 medium were purchased from Hyclone (Beijing, China). Leibovitz's L-15 medium was purchased from Gibco (CA, USA). For these mediums, 10% fetal bovine serum (Gibco, CA, USA) was added. A humidity incubator with 5% CO2 at 37°C was used to store these cells.

### 2.4. Quantitative Real-Time PCR (qRT-PCR)

Total RNA was acquired using TRIzol Reagent Kit (Invitrogen, USA), and cDNA was formed using PrimeScript RT Kit (TaKaRa, Dalian, China). Real-time PCR with 3 replicates in 20 *μ*l reactions using SYBR Reagent Kit (TaKaRa, Dalian, China). The inner reference was GAPDH and U6. The 2-*ΔΔ*Ct method was used to calculate the relevant ploidy changes in RNA expression. Application of primer pairs was as follows: mir-1266-3p F, 5′-CAATTATTCAAAAGATGCTTGGG-3′, mir-1266-3p R, 5′-GGTATGGCCCTCCAGGTTAA-3′; P4HA3 F, 5′−AAGTGGAGTACCGCATCAGC-3′, P4HA3 R, 5′−TTGGTGACGTAGCATGGTCAA-3′; U6 F, 5′-ATTGGAACGATACAGAGAAGATT-3′, U6 R, 5′-GGAACGCTTCACGAATTTG-3′; N-cadherin F, 5′-TCAGGCGTCTGTAGAGGCTT-3′, N-cadherin R, 5′-ATGCACATCCTTCGATAAGACTG-3′; snail F, 5′-CCCCAATCGGAAGCCTAACT-3′, snail R, 5′-GGTCGTAGGGCTGCTGGAA-3′; E-cadherin F, 5′-ACACCATCCTCAGCCAAGA-3′, E-cadherin R, 5′-CGTAGGGAAACTCTCTCGGT-3′; vimentin F, 5′-AAAACACCCTGCAATCTTTCAGA-3′, vimentin R, 5′-CACTTTGCGTTCAAGGTCAAGAC-3′; GAPDH F, 5′-ACTTTGGTATCGTGGAAGGACTC-3′, GAPDH R, 5′-GAGGCAGGGATGATGTTCTGG-3′.

### 2.5. Transfection

In order to detect the function of colon cancer cells in further, miR-1266-3p mimic, the control of miR-1266-3p mimic (miR-NC), the pcDNA-Flag-P4HA3 (Flag-P4HA3), and the pcDNA-Vector (Vector) were synthesized and purified by the General Biotechnology Co. (Chuzhou, China). Transfection performance under influence of Lipofectamine 3000 kit (Invitrogen, USA) was based on instruction manual. Cells were cultured for 48 h, collected, and assayed for transfection efficiency.

### 2.6. Dual-Luciferase Reporter Gene Test

P4HA3 was predicted as a target gene of miR-1266-3p by Targetscan (http://www.targetscan.org/). Wild and mutant 3'-UTRs of P4HA3 were designed and synthesized by General Biosystems Ltd (Chuzhou, China) and then embedded into PmirGLO plasmid. Co-transfection was performed in HEK-293 cells with Lipofectamine3000 kit (Invitrogen, USA). Relative luciferase activity of cells transfected for 48 hours as a child was determined using dual luciferase reporter system (Promega, USA).

### 2.7. Western Blot

Cells were lysed for 10 min at 4°C with adding RIPA lysis buffer (Thermo Fisher Scientific). The solution was then put into a 4°C centrifuge at 12000 r/min for 15 minutes. The amount of protein loaded in each line was about 40 *μ*g, and separated in the presence of 10% SDS-PAGE. Then, it was transferred to PVDF membrane. After that, the PVDF membrane was kept in 5% skimmed milk powder and held at room temperature for 1 hour. Then, the PVDF membrane was incubated overnight in the refrigerator at 4°C with primary antibody and incubated for 2 hours with secondary antibody. The antibodies were as follows: rabbit anti-P4HA3 (Biorbyt, orb77765), mouse anti-GAPDH (Abcam, ab9484), rabbit anti-E-cadherin (Abcam, ab18203), rabbit antivimentin (Abcam, ab217673), rabbit anti-N-cadherin (Abcam, ab76011), and rabbit antisnail (Abcam, ab216347).

### 2.8. Cell Counting Kit-8 (CCK-8) Assay

CCK8 kit was purchased from Beyotime Biotechnology (Shanghai, China). 1000 cells were added to each well of the 96-well plate and placed in a 5% CO2, 37°C incubator. For the first five days, every well was treated by 10ul CCK-8 reagent and then placed in a constant temperature of 37°C for 2 h. The final absorption was determined at 490 nm.

### 2.9. Colony-Forming Assay

After adding 3 ml of complete medium containing 10% fetal bovine serum to each well of the six-well plate, 500 cells were added to each well, and then, the six-well plate was stored at 5% CO2 and 37°C for 10-14 days until large clonal clusters were visible. Each well of the six-well plate was washed with PBS, fixed with 4% paraformaldehyde, and stained with 1% crystal violet. At last, the number of clonal clusters was counted.

### 2.10. Migration and Invasion Assay

The wound-healing assay was used to verify cell migration ability. Cells had been grown to confluence in cell culture medium with 10% serum. First, a waste wound was created with a 10 *μ*l pipette tip, and then, PBS was used to remove the waste and dead cells. The distances at 0 and 48 h were observed and recorded by using an inverted microscope. Six fixed fields of view were selected and relative migration distances were calculated.

A transwell invasion assay was used to study the role of miRNA-1266-3p in the invasion of colon cancer cells. 1 × 105 cells cultured in serum-free culture medium were seeded into a BD matrix gel-covered transwell insert. 48 hours later, the infiltrated cells at the bottom of the transwell chamber were fixated with 4% paraformaldehyde for a half hour before being stained with 1% crystal violet for a half hour. Then, the chamber was washed three times in PBS before taking pictures. Five separate views under high magnification were photographed, and the mean number of invaded cells per view was displayed in the figure.

### 2.11. Animal Experiments

Colon cancer cells (5 ×106) were injected hypodermically into the left epigastrium of the 4~5-week-old nude male BALB/c mice. Tumor sizes were measured every 3 days. The formula for calculating tumor volume was as follows: Tumor volume (mm^3^) = (Long axis × Short axis^2^)/2. The tumor tissues were removed after 25 days. In a metastasis experiment in vivo, nude mice were anesthetized, and the spleen was exposed by a left flank incision. After injecting 1×106 colon cancer cells into the spleen for one month, we euthanized the mice and collected liver samples to detect metastatic nodules.

### 2.12. Statistical Analysis

Information for the data was provided as mean ± SD for more than or equivalent to 3 independent trials. Graphpad Prism 8.0 (CA, USA) was used to analyze and graph the experimental data. Comparative analysis between groups was performed using Student *t*-test (two groups) or one-way ANOVA (multiple groups). The *P* value <0.05 was considered to be statistically significant.

## 3. Results

### 3.1. The Expression of miR-1266-3p Was Downregulated in Colon Cancer Tissue and Cell Lines

**Figure 1:**
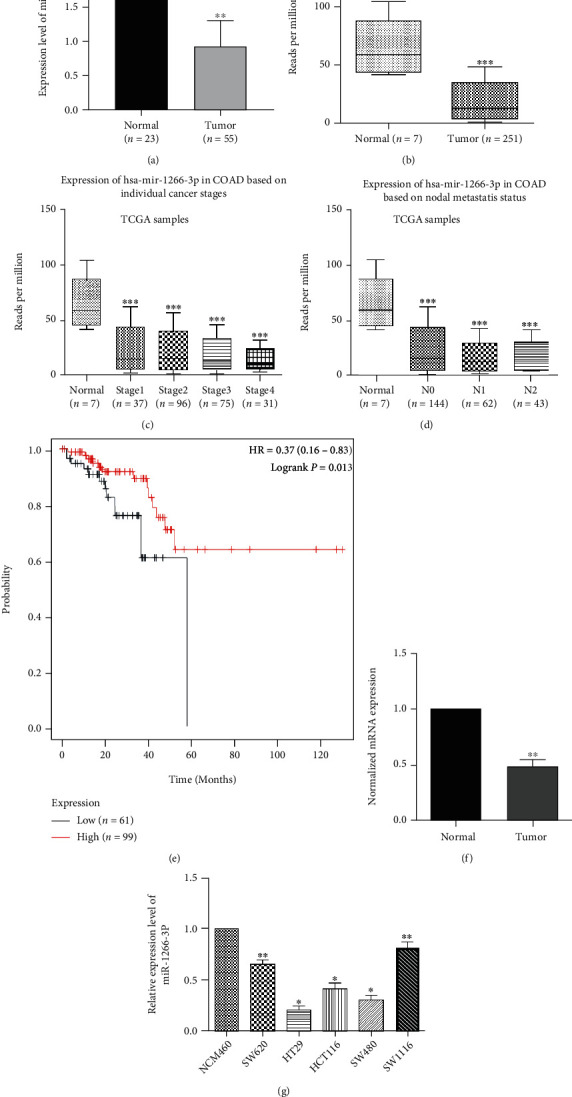
MiR-1266-3p expression in colon carcinoma cells and tissues. (a) MiR-1266-3p expression in the GSE35834 dataset of the GEO database (∗∗*P* < 0.01). (b) MiR-1266-3p expression in TCGA-COAD database (∗∗∗*P* < 0.001). (c) MiR-1266-3p expression in different stages of colon cancer (∗∗∗*P* < 0.001). (d) MiR-1266-3p expression in the different lymph node metastasis states in colon cancer (∗∗∗*P* < 0.001). (e) Kaplan-Meier survival curve comparison of miR-1266-3p differential expression in colon cancer. (f) MiR-1266-3p expression in five pairs of colonic carcinoma tissues and para-cancerous tissues (∗∗*P* < 0.01). (g) MiR-1266-3p expression in six types of cell lines (∗*P* < 0.05∗∗*P* < 0.01).

For determining the association of miR-1266-3p expression with colon cancer, we first analyzed miR-1266-3p expression in the GSE35834 dataset of the GEO database and the TCGA-COAD database. The result showed that miR-1266-3p expression of colon tumor tissue is obviously decreased than normal tissue next to cancer (Figures 1(a) and 1(b)). Naturally, based on the Ualcan online analysis software, we found that low miR-1266-3p expression negatively correlated with tumor stage and lymph node metastasis (Figures 1(c) and 1(d)). Next, we assessed the overall survival (OS) prognostic value of miR-1266-3p in TCGA-COAD database using Kaplan Meier plotter. The results showed decreased miR-1266-3p expression correlated remarkably to low OS in colon cancer (*P* = 0.013) (Figure 1(e)). For further validation of the above database findings, we first verified the expression levels of miR-1266-3p in five pairs of colon cancer tissues and paracancerous tissues by qRT-PCR. The expression of miR-1266-3p was significantly lower in colon cancer tissues than in paracancerous tissues in our results (Figure 1(f)). Immediately after, we used qRT-PCR to analyze miR-1266-3p expression in SW620, HCT116, HT29, SW480, and SW1116; 5 types of colon cancer cells; and NCM480 which was a kind of normal colonic epithelial cell. The results showed that miR-1266-3p expression in these five types of colon cancer cells was distinctly lower than normal colon cancer cells, particularly in SW480 and HT29 cells (Figure 1(g)).

### 3.2. P4HA3 Was a Direct Target of miR-1266-3p

**Figure 2:**
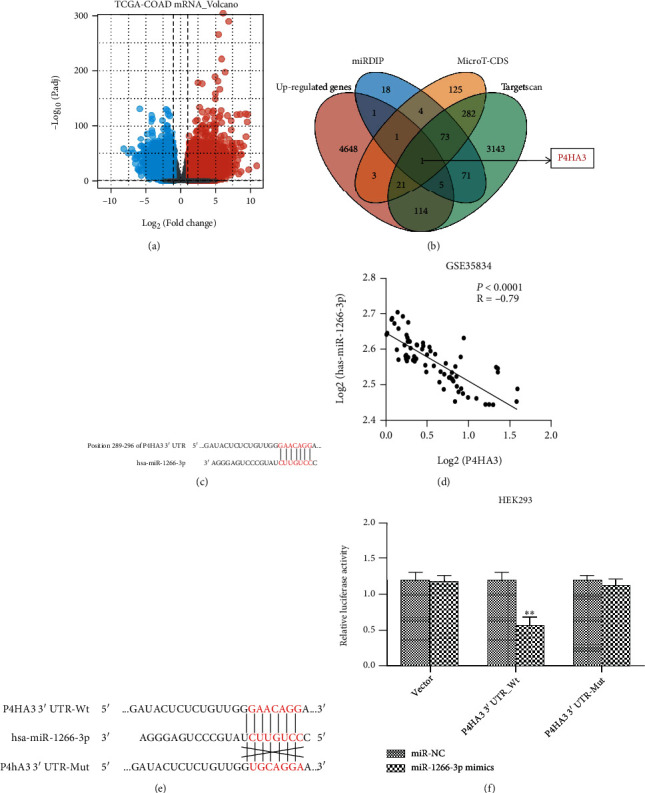
P4HA3 was a direct target of miR-1266-3p. (a) Volcano map of differentially expressed genes in normal and tumor groups. The red ball represents upregulated gene and the blue ball mean downregulated gene. (b) Venn diagram of upregulated genes and predicted target genes. (c) A schematic diagram of miR-1266-3p at the 3'-UTR predicted binding site of P4HA3 mRNA in the Targetscan database. (d) The correlation of miR-1266-3p with P4HA3 in the GSE35843 dataset was analyzed using the Pearson's method (*r* = −0.79, *P* < 0.0001). (e) Schematic diagram of the luciferase reporter vector construction for wild-type P4HA3 3'UTR (P4HA3 3'UTR-Wt) and miR-1266-3p-binding site mutation (P4HA3 3'UTR-Mut). (f) Luciferase reporter assay of HEK293 cells cotransfected with constructed luciferase reporter vectors (P4HA3 3'UTR-Wt, P4HA3 3'UTR-Mut, empty Vector) and miR-1266-3p mimics or negative control mimics (miR-NC) (∗∗*P* < 0.01).

In order to find the differentially expressed genes of colon cancer, we downloaded the TCGA-COAD database from GDC (https://portal.gdc.cancer.gov/), including 480 colon cancer samples and 41 normal samples. Because of the great number of differentially expressed genes gained from TCGA, the thresholds were set to *P*.adj < 0.05 and ∣log2(FC) | >2. 6431 genes were screened from TCGA-COAD, including 4886 genes upregulated and 1545 genes downregulated in expression (Figure 2(a)). Genes targeted by miR-1266-3p were analyzed by miRDIP (http://ophid.utoronto.ca/mirDIP/), MicroT-CDS (http://diana.imis.athena-innovation.gr/DianaTools/index.php?r=microT_CDS/index), and TargetScan (http://www.targetscan.org/vert_72/) databases and intersected with 4886 upregulated genes in the TCGA-COAD. One target gene (P4HA3) was finally obtained (Figure 2(b)). P4HA3 was characterized to be a potential target for miR-1266-3p by bioinformatics analysis using Tagetscan, based on P4HA3 3'UTR sequence of the putative target (Figure 2(c)). For further confirming correlation between miR-1266-3p and P4HA3, we downloaded the GSE35834 dataset from GEO, and this dataset contained the matched miRNA and gene expression data collected from 78 samples (23 normal tissues adjacent to cancer and 55 colon cancer tissues). Following an in-depth analysis of the GSE35834 dataset, It could be demonstrated that miR1266-3p expression in colon cancer tissues correlated remarkably negatively to P4HA3 expression (*r* = −0.79, *P* < 0.0001) (Figure 2(d)). In order to prove that P4HA3 can be targeted by miR-1266-3p in a direct manner, we constructed a fluorescent reporter vector with a miR-1266-3p binding site on the P4HA3 3'UTR. The targeting of miR-1266-3p to P4HA3 was demonstrated by cotransfection of miR-1266-3p with P4HA3 3'UTR reporter vector into HEK293 and detection of luciferase activity in HEK293 cell. Meanwhile, we performed point mutation of P4HA3 3'UTR to construct a mutant P4HA3 3'UTR fluorescent reporter plasmid (Figure 2(e)). MiR-1266-3p overexpression caused reduction of fluorescence activity in the wild type, whereas there was no significant change of fluorescence activity detected in the mutant UTR (Figure 2(f)).

### 3.3. Mir-1266-3p Inhibited Proliferation and Clone Formation of Colon Cancer Cells, and This Effect Could Be Alleviated by Overexpressing P4HA3

**Figure 3:**
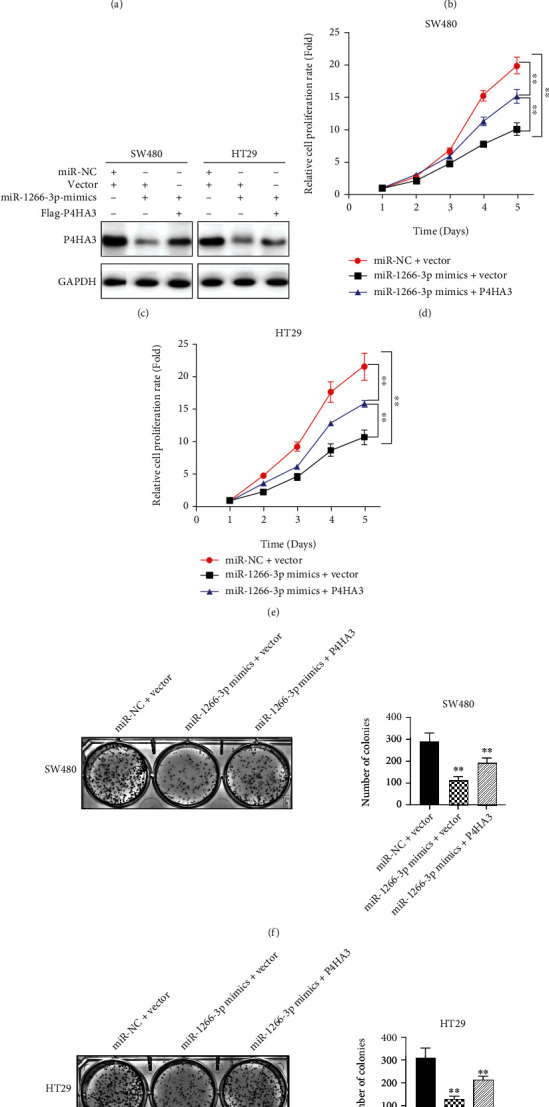
MiR-1266-3p inhibited proliferation and clone formation of colon cancer cells, and this effect could be alleviated by overexpressing P4HA3. (a–b) QRT-PCR indicated that miR-1266-3p expression increased and P4HA3 expression decreased after transfecting miR-1266-3p into SW480 and HT29 cells, whereas overexpression of P4HA3 could reverse the effect. (c) Western blot indicated that P4HA3 expression decreased after transfecting miR-1266-3p into SW480 and HT29 cells, whereas overexpression of P4HA3 could reverse the effect. (d–e) CCK8 assay revealed the reversal effects of P4HA3 overexpression on miR-1266-3p-mediated proliferative capacity of SW480 and HT29 cells (∗∗*P* < 0.01). (f–g) Colony-forming assay revealed the reversal effects of P4HA3 overexpression on miR-1266-3p-mediated clone formation ability of SW480 and HT29 cells (∗∗*P* < 0.01).

To further verify whether P4HA3 overexpression affects the effect of miR-1266-3p on the growth ability of colon cancer, we designed three groups in SW480 and HT29 cells, miR-NC+Vector group, miR-1266-3p mimics+Vector group, and miR-1266-3p+P4HA3 group, respectively. The miR-NC+Vector group was based on our cotransfection of SW480 and HT29 cells with miR-NC and empty vector. The miR-1266-3p mimics+Vector group was established when we cotransfected SW480 and HT29 cells with miR-1266-3p mimics and empty vector. The miR-1266-3p mimics+P4HA3 group was formed by cotransfecting SW480 and HT29 cells with miR-1266-3p mimics and Flag-P4HA3 plasmid. First, after transfection of miR-1266-3p mimics in SW480 and HT29 cells, the expression of miR-1266-3p was significantly higher at the mRNA level. However, the mRNA expression of P4HA3 was significantly decreased. But, the above effects were rescued when p4HA3 was overexpressed in both the two above-mentioned cells transfected with miR-1266-3p (Figures 3(a) and 3(b)). Then, we verified the decreased protein expression of P4HA3 in SW480 and HT29 cells after transfection with miR-1266-3p by using western blot. Interestingly, when P4HA3 was overexpressed in the above-mentioned two cells transfected with miR-1266-3p, the protein expression of P4HA3 was relatively elevated (Figure 3(c)). To evaluate the influence that miR-1266-3p has on colon cancer cell growth, we used CCK8 method to evaluate the growth curve. As shown in our results, there was a significant inhibition of the proliferation of SW480 and HT29 cells by miR-1266-3p overexpression. However, when P4HA3 was overexpressed in the cells with miR-1266-3p overexpression, the above effect was rescued (Figures 3(d) and 3(e)). Immediately after, we evaluated the colony-forming ability of miR-1266-3p in the aforementioned cells. The clonal clusters were stained, and at least 50 cells per clone were observed under high magnification. We got to know that miR-1266-3p overexpression resulted in reduced clone cluster size and number. Nevertheless, the above effects were rescued when P4HA3 was overexpressed in cells overexpressing miR-1266-3p (Figures 3(f) and 3(g)). Taken together, we confirmed that miR-1266-3p restrained colon cancer cell proliferation and clone formation, and overexpression of P4HA3 alleviated this effect.

### 3.4. P4HA3 Reversed the miR-1266-3p Mediated Capability of Inhibiting Cell Migration and Invasion in Colon Cancer Cells

**Figure 4:**
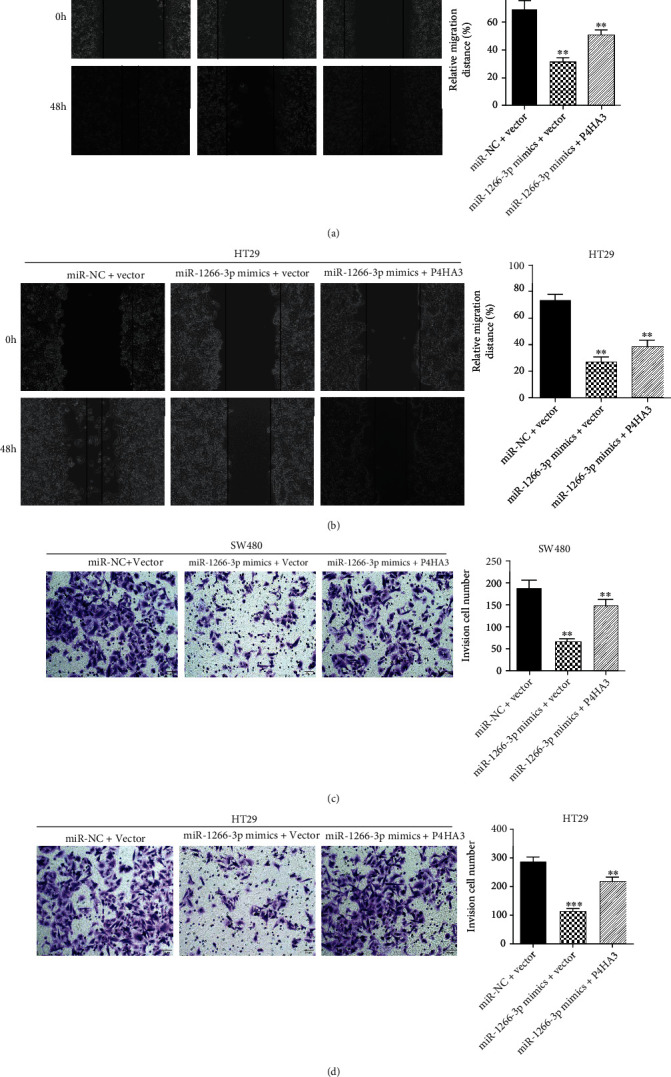
P4HA3 reversed the miR-1266-3p mediated ability of inhibiting cell migration and invasion in colon cancer cells. (a–b) The reversal effects of P4HA3 overexpression on miR-1266-3p-mediated migratory capacity of SW480 and HT29 cells was be verified by wound-healing assay (∗∗*P* < 0.01). (c–d) Tranwell assay revealed the reversal effects of P4HA3 overexpression on miR-1266-3p-mediated invasive capacity of SW480 and HT29 cells (∗∗*P* < 0.01, ∗∗∗*P* < 0.001).

To verify the effect of miR-1266-3p on the migratory ability of colon cancer cells and whether P4HA3 can reverse this effect, we performed wound scratching assays in SW480 and HT29 cells. In comparison to SW480 and HT29 cells expressing miR-NC, overexpressing miR-1266-3p showed reduced motility (Figures 4(a) and 4(b)). Furthermore, when P4HA3 was overexpressed in the above two types of cells overexpressing miR-1266-3p, the above-mentioned impaired motility could be rescued remarkably (Figures 4(a) and 4(b)). Subsequently, we assayed the effect of miR-1266-3p on the invasive ability of colon cancer cells using BD Matrigel invasion assay and whether P4HA3 could reverse this effect. As expected, the suppressive influence of miR-1266-3p on invasive ability could also be significantly reversed by P4HA3 (Figures 4(c) and 4(d)). In summary, P4HA3 reversed miR-1266-3p-mediated inhibiting migration and invasion of colon cancer cell.

### 3.5. MiR-1266-3p Inhibited EMT in Colon Cancer by Targeting P4HA3

**Figure 5:**
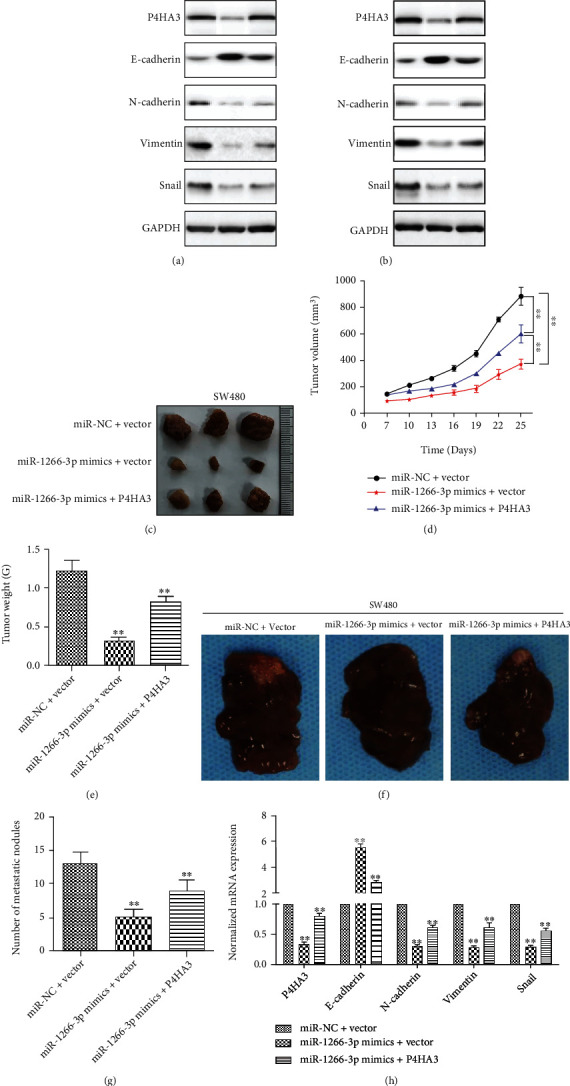
MiR-1266-3p inhibited EMT by targeting P4HA3 in colon cancer cells. (a) The reverse effect of P4HA3 overexpression on miR-1266-3p-mediated changes in EMT-associated proteins in SW480 cells by western blot. (b) The reverse effect of P4HA3 overexpression on miR-1266-3p-mediated changes in EMT-associated proteins by western blot in HT29 cells. (c–e) Tumor size was calculated every three days after tumor formation. The tumors were stripped and weighed after 25 days of injection (∗∗*P* < 0.01). (f–g) In vivo metastatic assay by spleen injection revealed miR-1266-3p suppressed colon cancer hepatic metastasis by targeting P4HA3 (∗∗*P* < 0.01). (h) Detection of P4HA3 and EMT-related markers expression in liver metastatic nodules by qRT-PCR (∗∗*P* < 0.01).

EMT is believed to have relevance to the ability of the cancer cells invasion and migration. Therefore, we tested the effects of miR-1266-3p on EMT-related proteins of colon cancer cells by using western blot. Our data suggested that miR-1266-3p overexpression resulted in downregulation of P4HA3 expression, as well as vimentin, snail, and N-cadherin expression downregulation and E-cadherin expression upregulation in SW480 and HT29 cells. However, following overexpression of P4HA3 in the above-mentioned two types of cells overexpressing miR-1266-3p, the expression of vimentin, snail, and N-cadherin increased, and the expression of E-cadherin decreased (Figures 5(a) and 5(b)). We conducted experiments in vivo by building xenograft mouse models in order to further verify the effects of miR-1266-3p on the growth, metastasis, and EMT of colon cancer. Tumor volume and weight of mice injected with SW480 cells overexpressing miR-1266-3p or P4HA3 were detected. Overexpression of miR-1266-3p reduced tumor growth rate, decreased tumor volume, and significantly reduced tumor weight compared to controls, whereas overexpression of P4HA3 dramatically reversed miR-1266-3p-induced decreases in tumor growth rate, volume, and weight (Figures 5(c)–5(e)). In addition, we discovered that the number of liver metastatic nodules was lower in mice injected with miR-1266-3p mimic-transfected SW480 cells compared to control mice, while P4HA3 overexpression significantly reversed the inhibition induced by miR-1266-3p in liver metastasis (Figures 5(f) and 5(g)). In addition, we detected the expression of P4HA3 and EMT-related markers in liver metastatic nodules using qRT-PCR. The results showed that miR-1266-3p overexpression resulted in downregulation of P4HA3 in liver metastatic nodules, as well as vimentin, snail, and N-cadherin expression downregulation and E-cadherin expression upregulation. However, P4HA3 overexpression could reverse the above effects induced by miR-1266-3p (Figure 5(h)). As a result, it was clear that miR-1266-3p inhibited EMT by targeting P4HA3 in colon cancer.

## 4. Discussion

As one of the most common malignant tumors in humans, colon cancer poses a serious threat to public health. Approximately 20% of patients show metastases at diagnosis, and approximately 30% of patients with stage II/III relapse within 5 years despite radical surgical treatment [25]. Hence, finding new diagnostic and therapeutic approaches and improving colon cancer patients' survival quality are the pressing issues for tumor research. In these years, modern biotechnology has advanced the vision of the diagnosis and treatment of cancer to genes or molecules. [26, 27].

The miRNAs are participating in nearly the entire range of vital activities, covering cell growth and death, organogenesis, somatogenesis, blood production, and lipid metabolism as well as other biological and pathological procedures [28–30]. In the recent years, with the advancement of molecular biology techniques, a growing number of researches have demonstrated that miRNAs are intimately linked to the developmental mechanisms of multiple cancers, including colon cancer [31–33]. As an important member of miRNAs, miR-1266-3p has been investigated in many types of tumors at present. There is a report that high miRNA-1266-3p expression in the liver cancer is associated with the poor prognosis of patients [34]. In another study, upregulating miR-1266-3p could strengthen the resistance of pancreatic cancer cells to gemcitabine chemotherapy [17]. Furthermore, it was also proved that miR-1266-3p inhibited the metastasis in papillary thyroid cancer cells by targeting FGFR2 [19]. Thus, it is clear that miR-1266-3p has different functions in different kinds of cancer, which can be either a tumor promoter or a tumor suppressor. Nevertheless, there has been no reported action of miR-1266-3p in colon cancer. For the first time, our work demonstrated the suppressive effect of miRNA-1266-3p on colon cancer proliferation, migration, and invasion.

Epithelial-mesenchymal transition (EMT) is the process by which the epithelial cell acquires mesenchymal characteristics and is associated with tumor growth, resistance to cancer therapy, and metastasis [35]. EMT contains many complex processes, such as poor intercellular junctions, acquisition of the capacity of invasion, and the expression of mesenchymal proteins [36, 37]. Tumor cells gain stem cell-like characteristics and therapeutic barriers during EMT, and the key process for epithelial cancer cells to obtain a malignant phenotype is the activation of EMT [38, 39]. There have been numerous studies proving that miRNAs are closely related to the EMT process of tumors. For example, miR-203a-3p could inhibit the epithelial-mesenchymal transition process in pancreatic cancer cells [40]. Another study demonstrated that miRNA-17-5p overexpression inhibited EMT process in colorectal cancer cells [41]. However, it remains unclear that whether miR-1266-3p regulates EMT processes in tumors, especially in colon cancer. Prolyl 4-hydroxylase subunit alpha 3 (P4HA3) has been known by people that it has a relationship with many kinds of human cancers. There have been several studies indicating that P4HA3 is highly expressed, as an oncogene, activated in tumors as well as promoting EMT [23, 24, 42]. Here, our team demonstrated, for the first time, that miR-1266-3p could inhibit EMT by suppressing the expression of P4HA3.

However, there were two main limitations in our study. First, we only analyzed the expression of miR-1266-3p in colon cancer tissues and paracancerous tissues in the TCGA database without further collection of specimens for validation, which might lead to the focus of our subsequent work to be done. The other is that the TCGA-COAD database showed a strong correlation between miR-1266-3p and lymph node metastasis, stage, and other related pathological parameters, and we would collect clinicopathological data in the next step to further validate the relationship between miR-1266-3p and clinicopathological parameters.

## 5. Conclusions

In summary, we clarified the inhibitory role of miR-1266-3p in human colon cancer for the first time in our work. Low miR-1266-3p expression tightly correlated with poor survival and prognosis in colon cancer patients. MiR-1266-3p had a target relationship with P4HA3, and its expression was negatively correlated. MiR-1266-3p functioned as a tumor suppressor that inhibited growth, metastasis, and EMT in colon cancer via downregulation of P4HA3. This provides a reliable laboratory evidence for clinical prognosis of colon cancer and builds a basis for better targeted therapy in the future.

## Data Availability

Upon reasonable request, datasets generated and/or analyzed in the course of this study may be obtained from the corresponding author.

## References

[1] Malki A., ElRuz R. A., Gupta I., Allouch A., Vranic S., Al M. A. (2021). Molecular mechanisms of colon cancer progression and metastasis: recent insights and advancements. *International Journal of Molecular Sciences*.

[2] Sung H., Ferlay J., Siegel R. L. (2021). Global cancer statistics 2020: GLOBOCAN estimates of incidence and mortality worldwide for 36 cancers in 185 countries. *CA: A Cancer Journal for Clinicians*.

[3] Weinberg B. A., Marshall J. L. (2019). Colon cancer in young adults: trends and their implications. *Current Oncology Reports*.

[4] Yang L., Wang S., Lee J. J. (2019). An enhanced genetic model of colorectal cancer progression history. *Genome Biology*.

[5] Guo H., Ingolia N. T., Weissman J. S., Bartel D. P. (2010). Mammalian microRNAs predominantly act to decrease target mRNA levels. *Nature*.

[6] Iorio M. V., Croce C. M. (2017). MicroRNA dysregulation in cancer: diagnostics, monitoring and therapeutics. A comprehensive review. *EMBO Molecular Medicine*.

[7] Hayes J., Peruzzi P. P., Lawler S. (2014). MicroRNAs in cancer: biomarkers, functions and therapy. *Trends in Molecular Medicine*.

[8] Rupaimoole R., Slack F. J. (2017). MicroRNA therapeutics: towards a new era for the management of cancer and other diseases. *Nature Reviews Drug Discovery*.

[9] Ali S. Z., Langden S., Munkhzul C., Lee M., Song S. J. (2020). Regulatory mechanism of MicroRNA expression in cancer. *International Journal of Molecular Sciences*.

[10] Zhang J. X., Song W., Chen Z. H. (2013). Prognostic and predictive value of a microRNA signature in stage II colon cancer: a microRNA expression analysis. *Lancet Oncology*.

[11] Zhang H., Wang Z., Ma R., Wu J., Feng J. (2018). MicroRNAs as biomarkers for the progression and prognosis of colon carcinoma. *International Journal of Molecular Medicine*.

[12] Zeng M., Zhu L., Li L., Kang C. (2017). miR-378 suppresses the proliferation, migration and invasion of colon cancer cells by inhibiting SDAD1. *Cellular & Molecular Biology Letters*.

[13] Xi X., Teng M., Zhang L., Xia L., Chen J., Cui Z. (2020). MicroRNA-204-3p represses colon cancer cells proliferation, migration, and invasion by targeting HMGA2. *Journal of Cellular Physiology*.

[14] Liu H., Ma L., Wang L., Yang Y. (2019). MicroRNA-937 is overexpressed and predicts poor prognosis in patients with colon cancer. *Diagnostic Pathology*.

[15] Hongdan L., Feng L. (2018). miR-3120-5p promotes colon cancer stem cell stemness and invasiveness through targeting Axin2. *Biochemical and Biophysical Research Communications*.

[16] Sun C. M., Zhang G. M., Qian H. N. (2019). MiR-1266 suppresses the growth and metastasis of prostate cancer via targeting PRMT5. *European Review for Medical and Pharmacological Sciences*.

[17] Wang J., Liu Y., Wang X. (2018). MiR-1266 promotes cell proliferation, migration and invasion in cervical cancer by targeting DAB2IP. *Biochimica et Biophysica Acta-Molecular Basis of Disease*.

[18] Zhang X., Ren D., Wu X. (2018). miR-1266 contributes to pancreatic cancer progression and chemoresistance by the STAT3 and NF-kappaB signaling pathways. *Molecular Therapy--Nucleic Acids*.

[19] Fu Y. T., Zheng H. B., Zhang D. Q., Zhou L., Sun H. (2018). MicroRNA-1266 suppresses papillary thyroid carcinoma cell metastasis and growth via targeting FGFR2. *European Review for Medical and Pharmacological Sciences*.

[20] Chen L., Lu M. H., Zhang D. (2014). miR-1207-5p and miR-1266 suppress gastric cancer growth and invasion by targeting telomerase reverse transcriptase. *Cell Death & Disease*.

[21] Gorres K. L., Raines R. T. (2010). Prolyl 4-hydroxylase. *Critical Reviews in Biochemistry and Molecular Biology*.

[22] Song H., Liu L., Song Z., Ren Y., Li C., Huo J. (2018). P4HA3 is epigenetically activated by Slug in gastric cancer and its deregulation is associated with enhanced metastasis and poor survival. *Technology in Cancer Research & Treatment*.

[23] Wang T., Wang Y. X., Dong Y. Q., Yu Y. L., Ma K. (2020). Prolyl 4-hydroxylase subunit alpha 3 presents a cancer promotive function in head and neck squamous cell carcinoma via regulating epithelial-mesenchymal transition. *Archives of Oral Biology*.

[24] Long R., Liu Z., Li J., Yu H. (2019). COL6A6 interacted with P4HA3 to suppress the growth and metastasis of pituitary adenoma via blocking PI3K-Akt pathway. *Aging (Albany NY)*.

[25] Shah M. A., Renfro L. A., Allegra C. J. (2016). Impact of patient factors on recurrence risk and time dependency of oxaliplatin benefit in patients with colon cancer: analysis from modern-era adjuvant studies in the adjuvant colon cancer end points (ACCENT) database. *Journal of Clinical Oncology*.

[26] Zaimy M. A., Saffarzadeh N., Mohammadi A. (2017). New methods in the diagnosis of cancer and gene therapy of cancer based on nanoparticles. *Cancer Gene Therapy*.

[27] Sun W., Shi Q., Zhang H. (2019). Advances in the techniques and methodologies of cancer gene therapy. *Discovery Medicine*.

[28] Cheng A. M., Byrom M. W., Shelton J., Ford L. P. (2005). Antisense inhibition of human miRNAs and indications for an involvement of miRNA in cell growth and apoptosis. *Nucleic Acids Research*.

[29] Martin E. C., Qureshi A. T., Dasa V., Freitas M. A., Gimble J. M., Davis T. A. (2016). MicroRNA regulation of stem cell differentiation and diseases of the bone and adipose tissue: perspectives on miRNA biogenesis and cellular transcriptome. *Biochimie*.

[30] Edelstein L. C., Bray P. F. (2011). MicroRNAs in platelet production and activation. *Blood*.

[31] Iqbal M. A., Arora S., Prakasam G., Calin G. A., Syed M. A. (2019). MicroRNA in lung cancer: role, mechanisms, pathways and therapeutic relevance. *Molecular Aspects of Medicine*.

[32] Khan A. Q., Ahmed E. I., Elareer N. R., Junejo K., Steinhoff M., Uddin S. (2019). Role of miRNA-regulated cancer stem cells in the pathogenesis of human malignancies. *Cell*.

[33] Yang Y., Meng W. J., Wang Z. Q. (2020). MicroRNAs in colon and rectal cancer - novel biomarkers from diagnosis to therapy. *Endocrine, Metabolic & Immune Disorders Drug Targets*.

[34] Huang X., Lai Y., Yao N. (2021). High expression of microRNA-1266 in hepatocellular carcinoma is associated with poor prognosis of patients and biological cell growth. *Oncology Letters*.

[35] Pastushenko I., Blanpain C. (2019). EMT transition states during tumor progression and metastasis. *Trends in Cell Biology*.

[36] Tzanakakis G., Kavasi R. M., Voudouri K. (2018). Role of the extracellular matrix in cancer-associated epithelial to mesenchymal transition phenomenon. *Developmental Dynamics*.

[37] Sun L., Fang J. (2016). Epigenetic regulation of epithelial-mesenchymal transition. *Cellular and Molecular Life Sciences*.

[38] Thiery J. P., Acloque H., Huang R. Y., Nieto M. A. (2009). Epithelial-mesenchymal transitions in development and disease. *Cell*.

[39] Polyak K., Weinberg R. A. (2009). Transitions between epithelial and mesenchymal states: acquisition of malignant and stem cell traits. *Nature Reviews Cancer*.

[40] An N., Zheng B. (2020). MiR-203a-3p inhibits pancreatic cancer cell proliferation, EMT, and apoptosis by regulating SLUG. *Technology in Cancer Research & Treatment*.

[41] Kim T. W., Lee Y. S., Yun N. H. (2020). MicroRNA-17-5p regulates EMT by targeting vimentin in colorectal cancer. *British Journal of Cancer*.

[42] Nakasuka F., Tabata S., Sakamoto T. (2021). TGF-beta-dependent reprogramming of amino acid metabolism induces epithelial-mesenchymal transition in non-small cell lung cancers. *Communications Biology*.

